# Crustal structure of La Palma Island inferred from 3D aeromagnetic modelling

**DOI:** 10.1038/s41598-025-34611-1

**Published:** 2026-01-10

**Authors:** María C. Romero-Toribio, Fátima Martín-Hernández, Juanjo Ledo

**Affiliations:** 1 Departamento de Física de la Tierra y Astrofísica, Facultad de Ciencias Físicas, Universidad Complutense de Madrid, Plaza de las Ciencias 1, Madrid, 28040 Spain; 2 Instituto de Geociencias, IGEO (CSIC-UCM), Calle Doctor Severo Ochoa 7, Madrid, 28040 Spain

**Keywords:** La Palma, Magnetic anomaly, Magnetic susceptibility, Geothermal system, Inversion, Crustal structure, Geophysics, Geomagnetism, Volcanology

## Abstract

La Palma, the second youngest island in the Canary archipelago, has been the subject of numerous geophysical studies. This study aims to use a new approach in the Island to unravel its crustal structure: 3D inversion of aeromagnetic anomalies. We analyze the magnetic anomaly map through standard data processing techniques and present the first 3D magnetic susceptibility model of La Palma. Our results suggest that the strongest magnetic signal in the North is attributed to the uplifted seamount dyke complex. The South of the island is characterized by three central bodies of high magnetic susceptibility up to 5 km depth comprising the bulk core material of the Cumbre Vieja volcano, in agreement with previous geophysical studies. We additionally identify lateral magnetic structures likely compatible with ancient volcanic structures that had not been inferred before. Both flanks of the Cumbre Vieja ridge are characterized by magnetic susceptibility lows compatible with hydrothermal alteration processes as previous studies suggested. This study demonstrates that the magnetic method and 3D magnetic susceptibility maps are valuable tools for initial geothermal and structural exploration in volcanic environments, such as La Palma, providing extensive spatial coverage and critical insights.

**Supplementary Information:**

The online version contains supplementary material available at 10.1038/s41598-025-34611-1.

## Introduction

The Canary Islands archipelago has been extensively studied for its volcanic activity, the risk of natural hazards, the interest of its geological history or its geothermal potential^1–3^. La Palma, one of the youngest islands, has been investigated through different geophysical methods, including gravimetry^4^, geodesy^4,5^, seismics^6,7^ and magnetotellurics^8^ to explore its inner structure. Recent studies in La Palma focus on the monitoring of the last eruption^9–13^, which occurred in 2021 at the Tajogaite volcano.

La Palma can be divided into two volcanic units: the Northern Volcanic Complex (NVC) and the Cumbre Vieja (CV) volcano to the South. According to previous studies, the largest high-density^4,14^ and resistive^8^ body characterized by high seismic wave velocity values^6^ was interpreted as the Pleistocene uplifted seamount, the oldest stage of the NVC. To the South, the youngest and only active part of the island, low-density structures^4,14^ were interpreted as high thermal anomalies or fractured zones on both sides of the main volcanic ridge of Cumbre Vieja, with high-density values along the ridge itself. Supporting this, the geoelectric model by Di Paolo et al.^8^ revealed low-resistivity anomalies in these low-density areas, compatible with clay alteration caps, suggesting the presence of hot rocks or heating fluids beneath Cumbre Vieja. In contrast, high-resistivity values were found along the ridge, associated with the high-density areas. Additionally, studies using geodetic techniques showed a clear subsidence in the southernmost area of the island likely originated by a thermal source^15^. Seismic studies interpret low-velocity zones to the South of the island as hydrothermal alteration zones linked to the geothermal system imagined by previous studies. High wave velocity was also found beneath the main volcanic ridge^6,7,16^.

Magnetic anomalies in the Canary Islands have been interpreted and analyzed both regionally offshore^17^ and at local scale in the islands of Fuerteventura and Lanzarote^18^, Gran Canaria^19,20^, Tenerife^21,22^, La Gomera^23^ and El Hierro^24^, revealing the lithospheric structure of the volcanic archipelago. However, magnetic anomalies in La Palma have not been analyzed in detail before. Only magnetization by means of paleomagnetic studies were carried out in the island due to the attractiveness of a volcanic setup that has recorded up to two inversions of the geomagnetic field^25^. Those studies have been specifically focused on the Pliocene seamount^26^ or with special emphasis in paleointensity determinations in the entire island^27–29^.

Magnetic anomalies are a widely used tool to map subsurface magnetic structures in volcanic settings^30^ as they mainly reflect contrasts in rock magnetic susceptibility^31^. The vectorial nature of magnetization allows magnetic anomalies to reveal directional information from both shallow and deep sources, while airborne surveys allow the exploration of inaccessible regions, including offshore areas^32^, providing critical insight into subsurface structures. Under certain conditions, they can also provide indirect evidence of geothermal activity^33,34^. Ferromagnetic minerals in volcanic rocks exhibit significant remanent magnetization, which tends to decrease as temperatures approach 300–400 °C, especially in minerals with low blocking temperatures. Also, magnetic susceptibility, the physical property of rocks inferred in this study, decreases significantly near the Curie temperature for ferromagnetic minerals, such as magnetite (580 °C), and more gradually for paramagnetic rock matrices^33^.

In this paper, we analyze the aeromagnetic anomalies of La Palma and construct the first 3D magnetic susceptibility model of the island prior to the 2021 eruption. This research aims to improve our understanding of the island’s crustal structure, offering valuable contributions to volcano-structural analysis and geothermal exploration.

## Geological setting

**Fig. 1:**
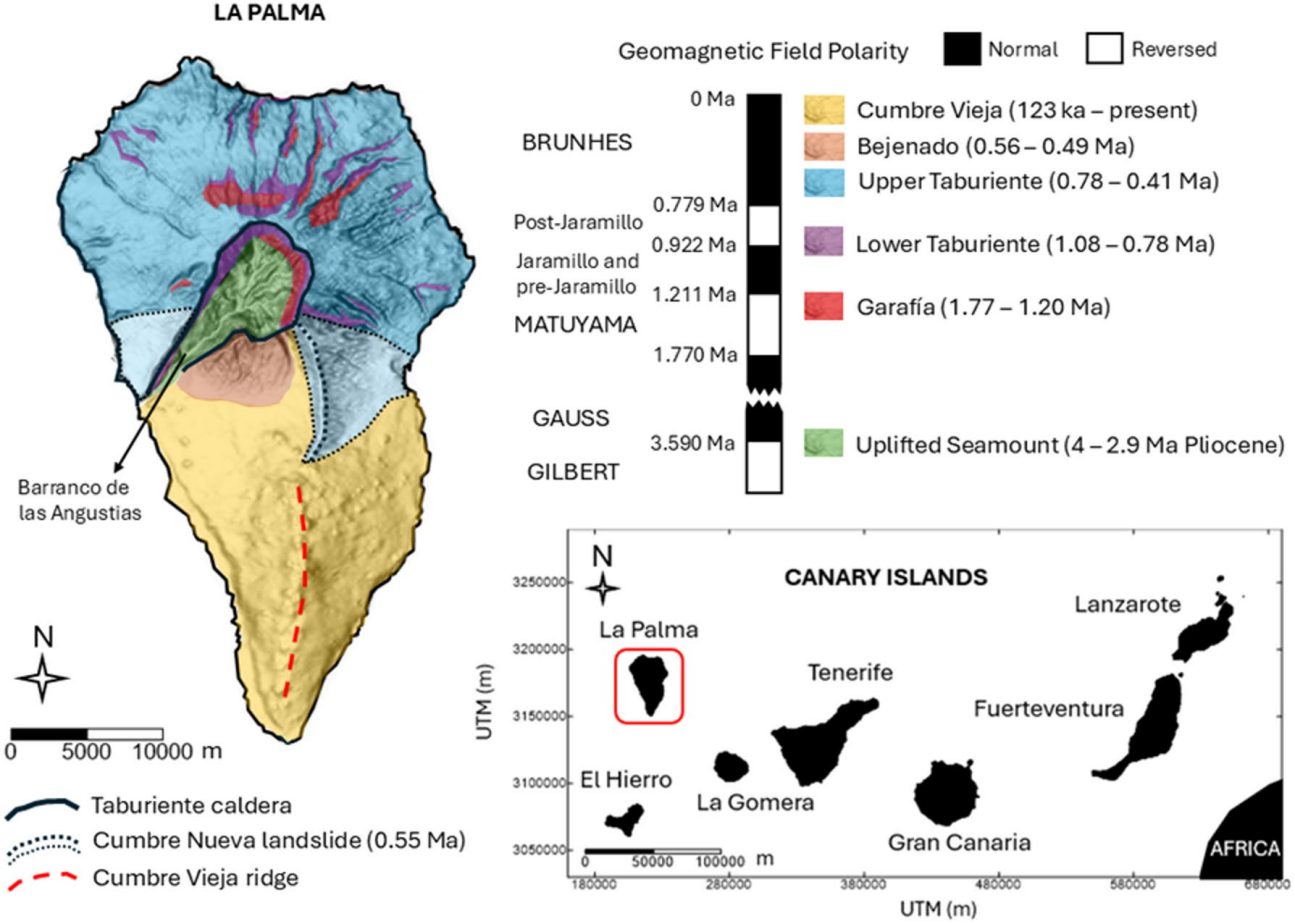
Geological map of La Palma. Ages summary from Carracedo et al.^35^. This figure is covered by the Creative Commons Attribution 4.0 International License.

La Palma is the second youngest volcanic island after El Hierro in the Canarian archipelago (Fig. 1). It is located 445 km away from Africa western coast and extends 47 km in the N-S direction and 28 km in the E-W direction. The highest peak is in the North of the island at 2426 m a.s.l. (above sea level) while the surrounding deep oceanic bottom is found at about 4000 m b.s.l. (below sea level)^35^. The island rests on the old Jurassic oceanic crust of the M25 magnetic anomaly^35^ and consists of two distinct volcanic units. To the North is the oldest volcanic sequence, overlying the uplifted Pliocene seamount that was formed 4 − 3 My ago^36^ during the reverse and normal polarity chrons (Gilbert and Gauss, respectively). As only occurs on the islands of Fuerteventura and La Gomera, on La Palma, the first submarine stage (Uplifted Seamount) of the volcanic island formation outcrops in the interior of the Taburiente caldera at about 500 m a.s.l. due to erosion. It is a plutonic complex intersected by a high density of basaltic dyke swarms, feeders of the subsequent volcanic sequence^35^. The subaerial stage of the island (2-0.4 Ma ago) started with the Garafía volcano emerging during the chron of reverse magnetic polarity Matuyama^37^. Approximately 1.2 Ma ago, after the gravitational collapse of southern Garafía, the formation of the Taburiente volcano began, lying above Garafía with a thickness of 1 km of highly homogeneous lavas. During its formation there are paleomagnetic records of the transition of the geomagnetic field from reverse polarity (Matuyama) to normal polarity (Bruhnes). Subsequently, during the current Brunhes chron, the Cumbre Nueva landslide and the Bejenado edifice occurred^37^.

The second volcanic unit of the island is located to the South and is known as Cumbre Vieja. It is elongated 17 km in the N-S direction and concentrates eruptive activity from the last 123 ka to the present day^38^. Its summit, reaching 1949 m a.s.l., is an alignment of fissures and eruptive vents that form a characteristic ridge zone (Fig. 1). It is composed by an extremely homogeneous alkaline sequence (alkaline basalts, basanites, trachybasalts, tephrites and phonolites) outcropping at the summit^35^ that are cooling under the present-day normal polarity chron (Brunhes).

## Methodology

### Magnetic data

**Fig. 2:**
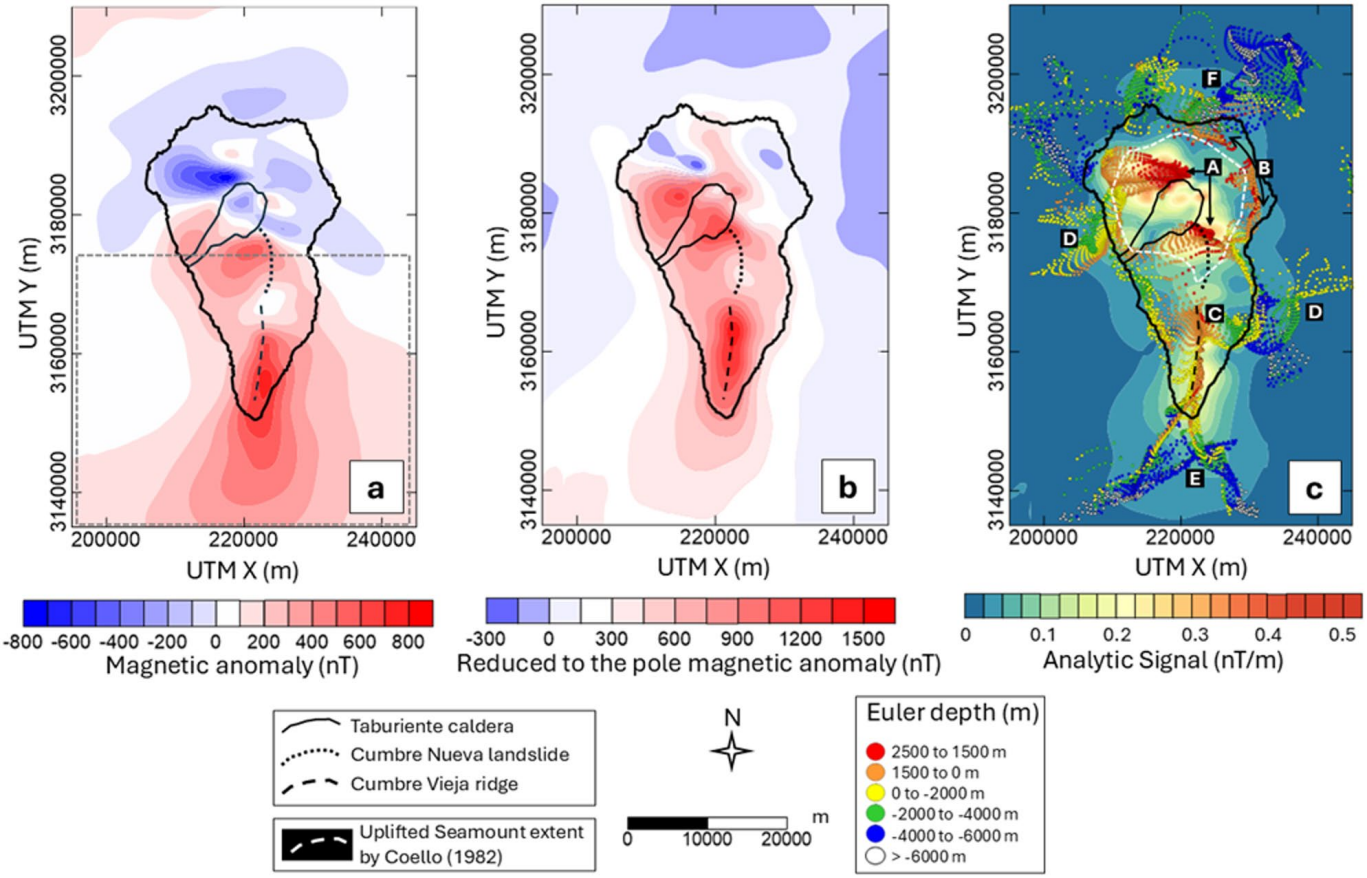
(**a**) Aeromagnetic anomaly map of La Palma acquired in 1993, from Socías and Mézcua^39^. The gray dashed line indicates the area where the total magnetization vector is assumed to be parallel to the present-day geomagnetic field. (**b**) Reduced to the pole magnetic anomaly map of La Palma. (**c**) Analytic Signal (contour map) and Euler solutions (coloured circles) in La Palma with a structural index of 1 and a window size of 10 km. Labels from “A” to “F” indicate the clusters of solutions interpreted in the discussion section. Negative values in depths indicate below sea level values. This figure is covered by the Creative Commons Attribution 4.0 International License.

The magnetic anomalies at La Palma were obtained during an aeromagnetic survey conducted by the Spanish National Geographic Institute in the Canary archipelago in 1993^39^. A scheme of the flight lines over the whole Archipelago is available in the Supplementary Figure S1. In La Palma, the flight lines were spaced 2.5 km in the N-S direction and 20 km in the E-W direction. After processing, they obtained a grid with a cell size of 2 km at an altitude of 3.2 km. The study area extends to 80 × 50 km^2^ and the magnetic anomalies range between − 800 nT to 800 nT (Fig. 2a). The Northern complex is characterized by an intense long wavelength dipolar anomaly, oriented according to an inducing geomagnetic field compatible to that of the acquisition time. Surrounding this anomaly are other dipolar anomalies of shorter wavelength and intensity whose polarity seems to be not so clear, probably influenced by the superposition of reversely magnetized lavas^25^. The South is mainly characterized by a large, markedly asymmetric dipolar anomaly in agreement with the present-day normal inducting geomagnetic field.

### Analysis of aeromagnetic anomalies through standard data processing techniques

After the qualitative analysis of the magnetic anomaly map of La Palma, we study it employing traditional potential field data techniques. Those mainly include the Reduction to the Pole (RTP) and Analytical Signal (AS) to determine the spatial location of sources, and the Standard Euler Deconvolution (ED) to determine the magnetic source depths^31^. The latter two allow us to decode the structure of the island using techniques independent of thermoremanence in the rocks. Therefore, by comparing them with the RTP map, we can locate areas where our magnetic susceptibility map should be interpreted with caution.

RTP is widely used to spatially locate sources from magnetic anomalies since it recovers maxima directly over the structures of interest^31^. However, the method assumes that magnetization is distributed homogeneously within the area. The most common scenario implies all magnetization to be parallel to the Earth Magnetic field or a non-parallel remanent magnetization to be relatively small. Therefore, the method usually uses the direction of the magnetic field at the time of data acquisition. In volcanic settings those assumptions are usually not happening^25,40^. In the Barranco de las Angustias at the NVC (Fig. 1), Koenigsberg ratios (Q; remanent/induced magnetization ratio) in gabbros, dikes, and subaerial lavas samples range from 1.72 to 18.69^26^. Values over 10 are indicative of a significant remanent magnetization. Moreover, the Northern volcanic sequence cooled during different magnetic field reversals (Fig. 1), thus, it is not advisable to reduce the anomalies to an average magnetization direction in this area, given such values of Q. In contrast, Cumbre Vieja (CV) was completely formed during the present Brunhes chron^35,41^. Thus, even if Q > 1, the total magnetization vector and both the induced and remanent magnetization vectors are parallel, making this simplification (assumed in our inversion) more accurate only here. The RTP (Fig. 2b) was calculated assuming the direction of the inducing magnetic field (IGRF model) at the time of the data acquisition (1993.8) at La Palma (I = 39.5º and D = -8.8º).

The 3D AS is another commonly used method in the interpretation of magnetic anomalies and has been successfully applied to volcanic environments^42–44^. This method, unlike RTP, relies on the vertical and horizontal gradients of the anomalies to show maxima over magnetization contrasts, regardless of the ambient magnetic field and source magnetization directions^45^. For the AS (Fig. 2c), the derivative of the magnetic field in the vertical direction was obtained by means of the Fast Fourier Transform.

The depth to the sources through the ED (circles in Fig. 2c) was estimated with a spatial window size of 10 km after testing 10 km, 20 km and 30 km. The deconvolution window size must be as small as possible to differ between close sources but large enough to allow the representation of broad anomalies arising from deep sources^46,47^. Thus, it must be at least twice the original data spacing and more than half the desired depth of investigation. The Structural Index (SI) is another parameter involved in the application of the ED method that is interpreted as a measure of the rate of change of a field with the distance^46^. Integer numbers 3, 2, 1 and 0 are associated with different geological shapes and we computed the depths for all SI. A SI of 1 is associated to a thin, infinitely deep dipping dyke or sheet edge (sill)^46^ better representing the delimiting contacts of the magnetic cores in La Palma when a particularly small window size of 10 km is used. The error tolerance in the solutions was limited to 8% of uncertainty, recovering high confidence results. To estimate the depth to the magnetic sources in La Palma though a different method we used the Radially Averaged Power Spectrum (RAPS)^31^ of the magnetic anomaly map. We applied the centroid method^48^ to the island’s anomalies to estimate the top, centroid and bottom of magnetic sources (Supplementary Fig. S2).

These methods allowed us to account for the remanence effects over the anomalies due to the superposition of lavas that underwent cooling during a period of reversed geomagnetic field. Subsequently, we determined the regions where the results of the magnetic inversion should be taken with caution, since the magnetic susceptibility is calculated based on the present-day geomagnetic field direction. Additionally, ED and the spectral analysis allow us to estimate the depth to the sources, used as initial constraints for our model.

### Magnetic inversion

ZondGM3D software (http://zond-geo.com/english/) was used to calculate the magnetic susceptibility model of La Palma. The 3D initial model mesh for the inversion consisted of 112 × 163 horizontal cells spaced 500 m and 13 vertical divisions increasing by a factor of 1.2, from 200 m a.s.l. for the subaerial part of the island to a bottom depth of 13 km b.s.l. By adding a relief to the initial mesh, the 3D model accounts for the topographic effects over the anomalies. The topography data used for the magnetic inversion belong to the digital elevation model gridded with a step of 25 m provided by the Spanish National Geographic Institute. The bathymetry was acquired from the ETOPO Global Relief Model from the National Oceanic and Atmospheric Administration (NOAA) with 15-arc-second geographic resolution. A background susceptibility value of 100 × 10^−4^ (cgs) has been included. This value represents the average susceptibility of basalts from literature standard values^49^. Additional depth constraints have been added based on Euler deconvolution and spectral analysis. The former shows that most of the sources in the South can be explained with solutions shallower than approximately 6 km b.s.l., and the RAPS indicates that the bottom to the sources is around 9.6 ± 2.8 km in La Palma, so sources deeper than that are not reliable. Based on this, we assume an initial 1D initial model that progressively decreases the magnetic susceptibility from 7 km to 13 km b.s.l., where the susceptibility was set to 0 (cgs). The direction of the inducing magnetic field at the time of the data acquisition (1993.8), an inclination of 39.5º and a declination of -8.8º, has also been included in the modelling. The inversion was performed by minimizing the L2 norm using the Occam method with a maximum of 15 iterations. The misfit of the model is available in Supplementary Fig. S3.

## Results

### Location and depth of magnetic sources in La Palma

The results obtained through the standard data processing techniques of the magnetic anomaly map revealed the location and depth of sources across the island. The RTP map in the Northern Hemisphere shows maxima directly over the magnetic sources^31^. Our RTP anomaly map (Fig. 2b) shows maxima to the South and East of the Taburiente caldera, and minima to the Northeast, whereas it shows a dominant and intense maximum along the ridge of Cumbre Vieja. The AS (Fig. 2c), which identifies the most pronounced contrasts in magnetic anomalies, reveals that the maxima are distributed heterogeneously across the Northern Complex and the Cumbre Vieja ridge volcanic unit. The standard ED solutions (circles in Fig. 2c) tend to cluster in areas of maximum anomaly contrasts, complementing the AS and providing an estimate of the depth at which the sources are located based on the gradients of anomalies in the three directions. A visual criterion was employed to select clusters of solutions with an SI of 1 (related to sill or dyke shapes) and a spatial window of 10 km. In the following we describe the main features of the Euler map, labelled from “A” to “F” in Fig. 2c. Two significant groups of solutions are observed above sea level (red and orange circles in Fig. 2c). One is associated with the Northern and Southern limits of the Taburiente Caldera (label “A”) and the other describes a circular shape with a large and irregular radius around the caldera (“B”). To the South of the island, from 1.5 km of altitude to about 2 km b.s.l. (orange- and yellow-coloured circles in Fig. 2c), the solutions show a linear tendency in the N-S direction (“C”) following the Cumbre Vieja ridge. Offshore to the West and East, from 2 to 6 km b.s.l. (green- and blue- coloured circles), we found two deeper sets of solutions (“D”). To the South and connected on land to the structure “C”, sources seem to split into two very deep offshore structures: to the Southeast and Southwest (“E”). To the North offshore, solutions are mostly concentrated in a small area reaching a great depth (“F”). The rest of the solutions seem to follow the coastlines right below the sea level. The spectral analysis over the entire island (RAPS, in Supplementary Figure S2) showed that the deepest sources are located around 9.6 ± 2.8 km, thus Euler solutions deeper than that (white circles in Fig. 2c) are considered not reliable.

### The 3D magnetic susceptibility model of La Palma

**Fig. 3:**
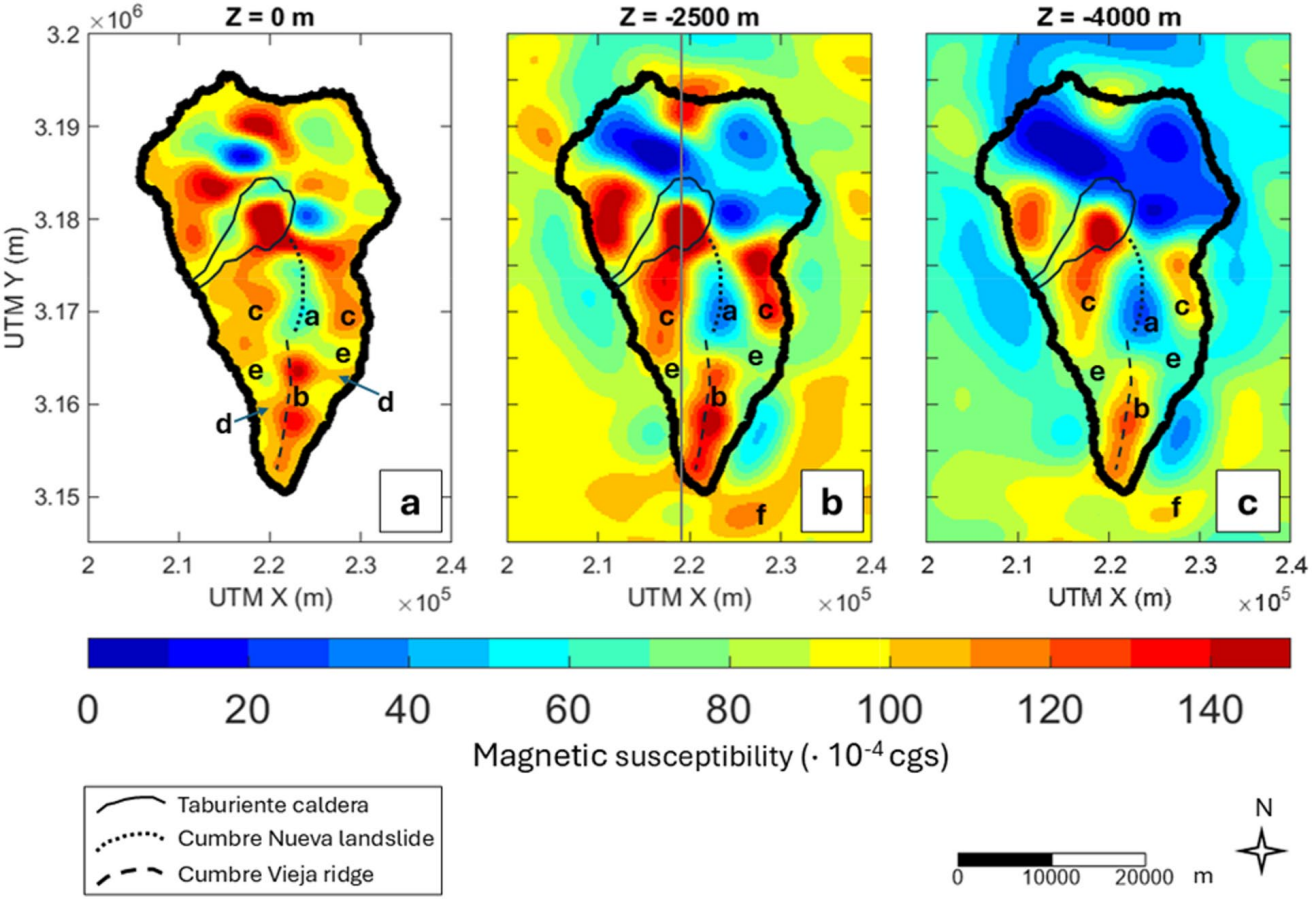
The 3D magnetic susceptibility model of La Palma at 0 m (sea level), 2.5 km b.s.l., and 4 km b.s.l. The coastlines are shown with a solid black line. Letters from **a–f** indicate the interpreted structural features. The gray solid line represents the cross section in Fig. 4.

We present our 3D magnetic susceptibility model of La Palma in Fig. 3. The Northern Volcanic Complex (NVC) shows a very heterogeneous magnetic susceptibility distribution, likely due to outcrops of remanent rocks from Garafía and Lower Taburiente edifices, cooled during the reverse polarity chron Matuyama^29,35,36^.

In Cumbre Nueva and Cumbre Vieja, where the direction of magnetization can be approximated to the present-day geomagnetic field, the magnetic susceptibility at all depths shows significant lateral gradients. There are two predominant structures here: one labelled as “a” with low magnetic susceptibility (≤ 60 × 10^− 4^ cgs), and another named “b”, elongated to the South in the N-S direction, with higher susceptibility (100–150 × 10^−4^ cgs). It is worth noting that “b” reveals the presence of three different magnetic lobes not detected before. We labelled “c” to another two high-susceptibility structures symmetrically found at both sides of “a” that persist up to 4 km b.s.l., slightly shallower than “b”. Shallower near the coastline (Fig. 3a), our model shows slightly high susceptibility areas (100–110 × 10^− 4^ cgs) labelled “d”. Another notable feature that persists at all depths is the presence of minima “e” with low magnetic susceptibility (60–90 × 10^− 4^ cgs), to the West and East of the volcanic ridge. Finally, we have assigned “f” to a high magnetic susceptibility (110–120 × 10^− 4^ cgs) body offshore that seems to be connected to “b” at depth.

## Discussion

### Structural features of La Palma from the aeromagnetic anomalies analysis

The magnetic anomaly map of La Palma shows similar intensities as those from neighbouring islands such as Tenerife^22^ or Gran Canaria^24^ but the features reveal a complex pattern (Fig. 2a). The map shows anomalies in the Northern Volcanic Complex (NVC) influenced by geomagnetic polarity transition records^25,26,40,41^.

We compared the Reduction to the Pole (RTP) anomaly map (Fig. 2b) which depends on the total magnetization direction, to the Analytic Signal (AS, Fig. 2c), not influenced by such direction to reveal magnetic sources. To the North, while the RTP shows an asymmetric shape with a maximum positive anomaly southwest of Taburiente, the AS (Fig. 2b) shows heterogeneous and concentric maxima around the Caldera de Taburiente. The discrepancy directly indicates the strong influence on the anomaly map of natural remanent magnetization in volcanic lavas from Lower Taburiente and Garafía edifices which cooled during periods of reversed magnetic field^50^ and have a Königsberger ratio greater than 10^26^. In comparison, Cumbre Vieja (CV) cooled entirely under the current normal polarity magnetic field (Brunhes chron) and therefore the remanent and induced magnetization vectors are parallel. RTP properly corrects the anomalies with the angle of the total magnetization vector and both maps agree in the location of the main magnetic sources.

Maxima in the AS seem to be related to the Uplifted Seamount complex, concentrated around the Caldera de Taburiente with the most magnetic material. The plutonic complex associated to the Uplifted Seamount has been previously imaged as a relatively circular structure around the Caldera de Taburiente^4,6,8^. It is characterized by a high density of dyke swarms and is therefore expected to have a strong magnetization compared to the surrounding material, such as basaltic coladas and pyroclasts^49^. The seamount stage is also exposed on the islands of La Gomera and Fuerteventura. Most of the magnetic signal mapped on La Gomera has been attributed to intense magnetization of intrusive complexes (plutonic bodies and dyke complexes) that represent this initial growth stage^23^. Additionally, in La Palma both the caldera and the Barranco de las Angustias, formed from the collapse of the former^35^, do not show maxima in the AS following the steep topography as in this area the magnetic material is missing.

Magnetic source location and depth estimations were obtained through the standard Euler deconvolution^46^ (circles in Fig. 2c) regardless of the remanent magnetization direction. The geological interpretation of Euler depths must be taken with caution since the structural index and window size of application is subjectively chosen by the authors^46^. Results should always be verified and interpreted considering prior knowledge from other independent techniques^46^. In our Euler depth map (Fig. 2c), we suggest that “A” is probably related to the strong contrasts in magnetic anomalies caused by the topography of the Caldera de Taburiente. The irregular concentric structure “B” is interpreted as the boundary between the Uplifted Seamount structure and its surroundings, providing insights into the extent of this intrusive complex as previously inferred by Coello^51^ (white dashed outline in Fig. 2c). A structure of high S-waves velocity with a similar extension was inferred before^6^. However, some discrepancies arise when comparing this extent with other geophysical studies that refer to a smaller resistive^8^ and dense^4^ core. In CV, Euler solutions (“C” in Fig. 2c), in agreement with the only maximum shown in the AS and the RTP, coincides with the N-S alignment of volcanic cones constituting the main magnetic structure of this area. Di Paolo et al.^8^ also imaged in this area a high-resistivity elongated structure up to 4.5 km depth. This N-S feature is clearly shown in our magnetic susceptibility model as a maximum in this property up to 5 km depth (labelled “b” in Fig. 3). Between 2 km and 6 km b.s.l., some features are revealed offshore (“D”). They could be related debris avalanche complexes mapped with sonar between 1 km and 3–4 km depth^52^. The magnetic shipborne study offshore^17^ recovered Euler solutions around La Palma but were attributed to deeper sources due to larger cell size (3.8 km) in their magnetic anomaly grid. The solutions labelled as “E” in Fig. 2c are very deep sources, showing some values that can be discarded according to the spectral analysis (9.6 ± 2.8 km). However, we should consider that previous studies evidenced the existence of a seamount^52^ and anomalous Bouguer^53^ and magnetic^17^ features from the South of La Palma extending nearly 27 km South eastwardly. Additionally, the model presented in this study shows an offshore maximum in magnetic susceptibility, labelled “f” in Fig. 3, reinforcing the hypothesis that there is a magnetic source located offshore to the southeast of La Palma. “F” shows similar depth values as in “E”. These Euler solutions are likely influenced by edge effects although some similitudes are found with the analytic signal of magnetic anomalies by Catalán et al.^17^.

### Magnetic susceptibility in La Palma

The 3D magnetic susceptibility model from this study is presented in Fig. 3. Bodies labelled “b” and “c” seem to be related to the emplacement of rocks cooled during the early period of Cumbre Vieja, specifically with the evolution of the volcano-structural lineament features. As previously suggested by the standard techniques applied to the anomaly map (AS, RTP and ED), according to the model, the primary source of the magnetic anomalies is a series of high magnetic susceptibility bodies (“b”) elongated in the N-S direction beneath the mountain ridge reaching depths of 5 km b.s.l. These structures, based on their high magnetic susceptibility and location (high density of vents), are interpreted as dyke intruded complexes or solidified magma chambers (plutonic bodies) that originated the oldest eruptions in this area. They are characterized by a high content of probably unaltered magnetic materials forming the magnetic core of CV.

**Fig. 4:**
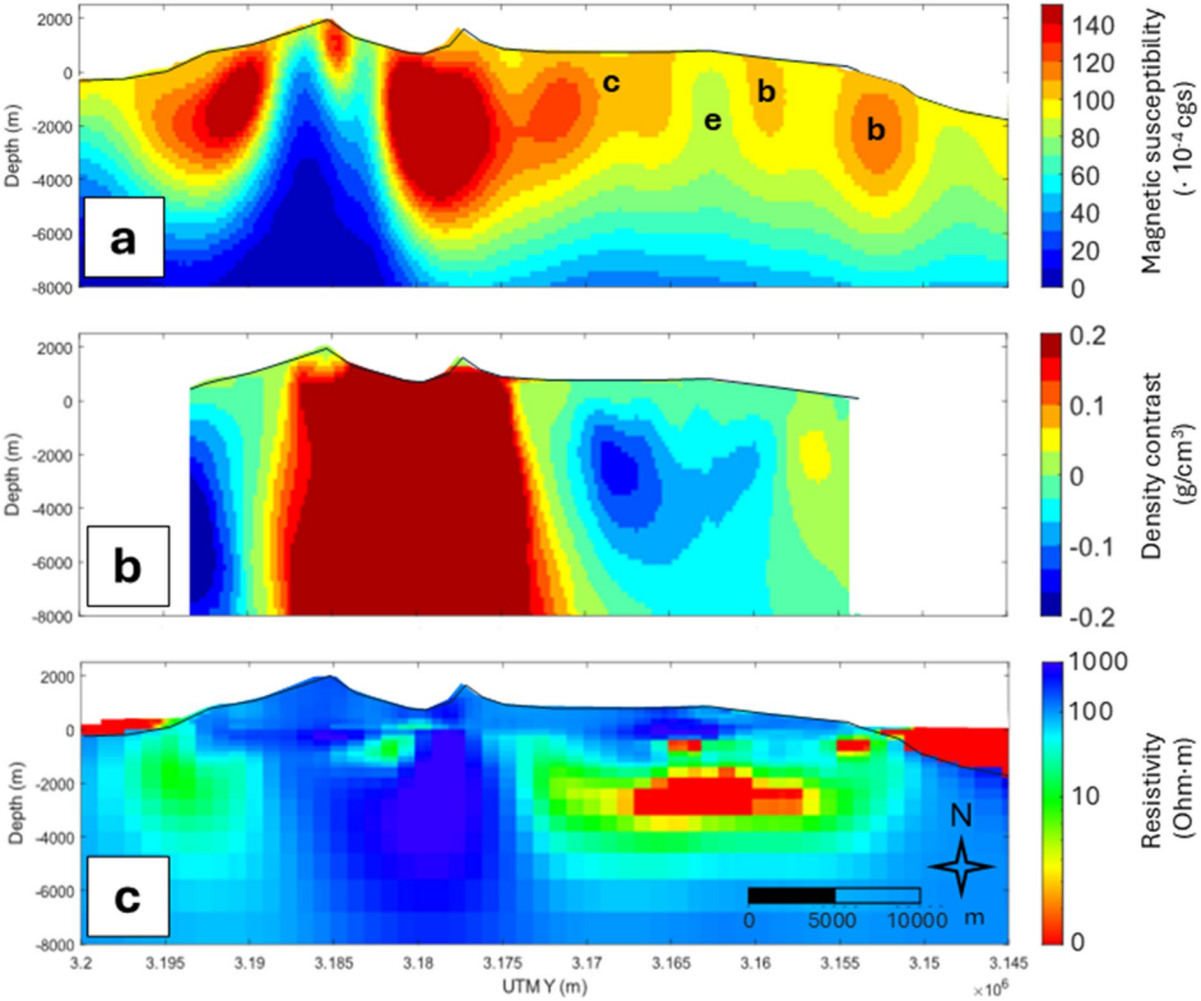
Cross sections (X = 219000 m) from (**a**) the magnetic susceptibility model of this study, where the letters **b**, **c** and **e** stand for the same bodies as those in Fig. 3, (**b**) the density contrast model from the joint inversion presented in our Supplementary Materials, and (**c**) the apparent resistivity model by Di Paolo et al.^8^. Topography and bathymetry are shown with a solid black line. This figure is covered by the Creative Commons Attribution 4.0 International License.

To allow an interdisciplinary interpretation of La Palma internal structure we additionally performed a joint magnetic and gravimetric inversion of La Palma using publicly available gravimetric data from Montesinos et al.^12^ and requested data from the Spanish National Geographic Institute (details on data and joint inversion are found in Supplementary Text and Supplementary Fig. S4). Note that, to enable the calculation of the cross-gradients of both potential fields, the joint inversion led to a decrease in the amount of data respect to the standalone magnetic inversion but still represents the same features with lower horizontal resolution. The density contrast model derived from the joint model (Fig. 4b and Supplementary Fig. S4) reveals very similar features to that published by other authors^4,9^: high-density values right below the volcanic ridge, consistent with the proposed structures from the magnetic model. The geoelectric model of La Palma shows a similar elongated structure with high electrical resistivity extending to nearly 5 km in depth^8^. The S-wave seismic tomography model also shows a high-velocity zone between 2 km b.s.l. and 5 km b.s.l. corresponding to the same structure^6^.

The Southern body of “b” at sea level is characterized by slightly lower susceptibility than the rest of the bodies in this structure and low density (Supplementary Fig. S4). This feature can be associated with the volcanic complex that produced the San Antonio eruptions in 1677 and Teneguía in 1971. As the lava flows and lapilli samples from La Palma are predominantly multi-domain^54^, we can assume that there are no Hopkinson peaks (leading to a susceptibility increase) near the Curie temperature. Therefore, the hotter material, without reaching the Curie temperature, becomes slightly less susceptible to magnetization. In agreement to this, the area hosts a hot spring complex (Fuente Santa) hosting a temperature around 40 °C^55^. This body also extents up to 4.5 km depth suggesting that the structure is also a substantial part of the bulk of Cumbre Vieja ridge.

The lateral structures labelled as “c” in Fig. 3, not inferred before by other studies, along with “b” could be associated with the oldest magmatic activity dated in this volcanic complex. The oldest eruptions (125 ka – 80 ka) that occurred in Cumbre Vieja are known as “Cliff-forming eruptions” and constitute the bulk of this volcano^35^. The outcrops of the products of these eruptions predominantly follow a system of three rifts oriented towards the Northwest, Northeast, and the South of the island. We interpret these bodies as the cause of this eruptive trend, constituting older magmatic cores from which magmatism in Cumbre Vieja has developed. Although the resistivity in this area is not remarkably high, as it might be expected, the existence of these structures is consistent with the magnetotelluric model of Di Paolo et al.^8^. The low resistivity structures observed on the Western and Eastern flanks of the Cumbre Vieja ridge (Supplementary Fig. S5), associated with clay caps, seems to be shaped and defined by the presence of other materials with different geoelectrical characteristics to the North, which could correspond to our “c” structures. The low magnetic susceptibility and high-density structure “a” has been associated with the Cumbre Nueva landslide scar. The neighbouring island of El Hierro also show very low magnetizations characterising the areas affected by recognized landslides^24^.

Bodies labelled “d” in Fig. 3 are characterized by substantial magnetic susceptibility, but not as intense as the previous. These bodies are also shallower, reaching only the first 2 km b.s.l. Given their susceptibility, shape, and geographical coincidence with the historical lava flow patterns of Tahuya (1585), San Juan (1949), Martín (1645), San Antonio (1677), or Teneguía (1971), these structures have been interpreted as the accumulation of highly consolidated volcanic products from recent eruptions.

Low susceptibility signatures “e” to both flanks of CV ridge in Fig. 3 are compatible with the effect of hydrothermally altered materials, high temperature areas or hot fluids^33,56^, supporting the hypothesis of an active geothermal system that was stated in previous studies^4,6,8,16^. Figure 4 shows the vertical N–S section (dashed line in Fig. 3b) of the magnetic model in comparison to those of the gravimetric joint model (Supplementary Fig. S4) and the geoelectric model from Di Paolo et al.^8^ (Supplementary Fig. S5). The three sections show clear agreement in the location of the geothermal system. However, the magnetic method does not show a clearly defined structure. This is due to the inherent limitations of the magnetic method, which is more effective at detecting shallow sources. The depth of the reservoir, together with the non-uniqueness of solutions from the potential methods inversion, results in a less well-defined structure and depth, although the low magnetic susceptibility signature, a characteristic of geothermal systems, is still present in the magnetic model.

The body labelled “f” in Fig. 3 is another volcanic structural lineament highlighted by our magnetic susceptibility model. This body coincides with the southeast-trending lineament feature from the offshore Euler solutions discussed earlier in this paper. Therefore, “f” is the offshore continuation (seamount) of the bodies conforming the bulk material under Cumbre Vieja. Our study provides the first geophysical imaging of this structure.

## Conclusion

We use standard data processing methods to analyze the aeromagnetic anomaly map of La Palma Island. Our findings reveal that most of the magnetic signal is linked to the Uplifted Seamount dyke complex in the North and to elongated bulk magnetic materials beneath the Cumbre Vieja ridge in the South. This analysis enabled us to account for the thermoremanence effect on the anomaly map, leading us to exclude the Northern Volcanic Complex from further discussion. Consequently, our study introduces the first 3D magnetic susceptibility model of La Palma. The South of the island is characterized by three central lobes of high magnetic susceptibility extending to depths of 5 km b.s.l. along the Cumbre Vieja ridge. The southernmost of these is clearly associated with historic volcanic activity. The model also revealed the existence of previously unidentified ancient volcanic structures, in addition to shallower structures associated with volcanic lava flows. The active geothermal system on the Eastern and Western flanks of Cumbre Vieja is characterized by substantially low magnetic susceptibility values at similar depths in agreement with previous geophysical studies.

This study demonstrates that the magnetic method is a valuable tool for imaging volcanic structures, enhancing our understanding in areas where other geophysical surveys are not feasible. Moreover, 3D magnetic susceptibility mapping can serve as a useful preliminary approach to complement other geophysical techniques in the search for geothermal resources.

## Supplementary Information

Below is the link to the electronic supplementary material.

Supplementary Material 1

## Data Availability

The magnetic susceptibility and density models generated during the current study are available in the Zenodo repository (10.5281/zenodo.14793827).

## References

[1] Piña-Varas, P. et al. 3-D magnetotelluric exploration of Tenerife geothermal system (Canary Islands, Spain). *Surv. Geophys.* **35**, 1045–1064 (2014).

[2] Rodríguez, F. et al. Surface geochemical and geophysical studies for geothermal exploration at the Southern volcanic rift zone of Tenerife, Canary Islands, Spain. *Geothermics* **55**, 195–206 (2015).

[3] Ledo, J. et al. 3D electrical resistivity of Gran Canaria Island using magnetotelluric data. *Geothermics* **89**, 101945 (2021).

[4] Camacho, A. G. et al. Structural results for La Palma Island using 3-D gravity inversion. *J. Geophys. Res. Solid Earth* 114 (2009).

[5] Escayo, J. et al. Geodetic study of the 2006–2010 ground deformation in La Palma (Canary Islands): observational results. *Remote Sens.* **12**, 2566 (2020).

[6] Cabrera-Pérez, I. et al. Geothermal and structural features of La Palma Island (Canary Islands) imaged by ambient noise tomography. *Sci. Rep.* **13**, 12892 (2023). 37558726 10.1038/s41598-023-39910-z PMC10412587

[7] Serrano, I., Dengra, M., Almendros, F., Torcal, F. & Zhao, D. Seismic anisotropy tomography beneath La Palma in the Canary islands, Spain. *J. Volcanol. Geotherm. Res.* **107870** (2023).

[8] Di Paolo, F. et al. La Palma Island (Spain) geothermal system revealed by 3D magnetotelluric data inversion. *Sci. Rep.* **10**, 18181 (2020). 33097774 10.1038/s41598-020-75001-z PMC7585421

[9] Fernández, J. et al. Shallow magmatic intrusion evolution below La Palma before and during the 2021 eruption. *Sci. Rep.* **12**, 20257 (2022). 36509802 10.1038/s41598-022-23998-w PMC9744821

[10] Blanco-Montenegro, I. et al. Volcanomagnetic signals related to the 2021 Tajogaite volcanic eruption in the cumbre Vieja rift (La Palma, Canary Islands). *J. Volcanol. Geotherm. Res.* **108200** (2024).

[11] De Luca, C. et al. Pre-and co‐eruptive analysis of the september 2021 Eruption at Cumbre Vieja Volcano (La Palma, Canary Islands) Through DInSAR measurements and analytical modeling. *Geophys. Res. Lett.* **49**, e2021GL097293 (2022).

[12] Montesinos, F. G. et al. Insights into the magmatic feeding system of the 2021 eruption at Cumbre Vieja (La Palma, Canary Islands) Inferred from Gravity Data Modeling. *Remote Sens.* **15**,1936 (2023).

[13] Piña-Varas, P. et al. Volcanic monitoring of the 2021 La Palma eruption using long-period magnetotelluric data. *Sci. Rep.* **13**, 15929 (2023). 37741929 10.1038/s41598-023-43326-0 PMC10517953

[14] Fernández, J. et al. Detection of volcanic unrest onset in La Palma, Canary Islands, evolution and implications. *Sci. Rep.* **11**, 1–15 (2021). 33510383 10.1038/s41598-021-82292-3 PMC7844277

[15] Prieto, J. F. et al. Geodetic and structural research in La Palma, Canary Islands, spain: 1992–2007 results. *Pure Appl. Geophys.* **166**, 1461–1484 (2009).

[16] Ortega-Ramos, V. et al. Evidence of a low‐velocity zone in the upper mantle beneath Cumbre Vieja volcano (Canary Islands) through receiver functions analysis. *Geophys. Res. Lett.* **51**, e2023GL105487 (2024).

[17] Catalán, M., Martín Davila, J. & Group, Z. W. A magnetic anomaly study offshore the Canary Archipelago. *Geophysics Canary Islands: Results Spain’s Exclusive Economic Zone Program* 129–148 (2005).

[18] Blanco-Montenegro, I., Montesinos, F., García, A., Vieira, R. & Villalaín, J. Paleomagnetic determinations on Lanzarote from magnetic and gravity anomalies: Implications for the early history of the Canary Islands. *J Geophys. Res. Solid Earth* **110** (2005).

[19] Blanco-Montenegro, I., Torta, J. M., García, A. & Araña, V. Analysis and modelling of the aeromagnetic anomalies of Gran Canaria (Canary Islands). *Earth Planet. Sci. Lett.* **206**, 601–616 (2003).

[20] Blanco-Montenegro, I., Montesinos, F. G. & Arnoso, J. Aeromagnetic anomalies reveal the link between magmatism and tectonics during the early formation of the Canary Islands. *Sci. Rep.* **8**, 42 (2018). 29311714 10.1038/s41598-017-18813-w PMC5758788

[21] Araña, V. et al. Internal structure of Tenerife (Canary Islands) based on gravity, aeromagnetic and volcanological data. *J. Volcanol Geotherm. Res.* **103**, 43–64 (2000).

[22] García, A. et al. High resolution magnetic anomaly map of Tenerife, Canary Islands. *Ann. Geophys.* (2007).

[23] Blanco-Montenegro, I., Montesinos, F. G., Nicolosi, I., Arnoso, J. & Chiappini, M. Three‐dimensional magnetic models of La Gomera (Canary Islands): Insights into the early evolution of an ocean island volcano. *Geochem. Geophys. Geosyst.* **21**, e2019GC008787 (2020).

[24] Blanco-Montenegro, I., Nicolosi, I., Pignatelli, A. & Chiappini, M. Magnetic imaging of the feeding system of oceanic volcanic islands: El Hierro (Canary Islands). *Geophys. J. Int.* **173**, 339–350 (2008).

[25] Tauxe, L., Staudigel, H. & Wijbrans, J. R. Paleomagnetism and 40Ar/39Ar ages from La Palma in the Canary Islands. *Geochem. Geophys. Geosyst.* 1 (2000).

[26] Gee, J., Staudigel, H., Tauxe, L., Pick, T. & Gallet, Y. Magnetization of the La Palma seamount series: implications for seamount paleopoles. *J. Geophys. Res. Solid Earth*. **98**, 11743–11767 (1993).

[27] Valet, J. P. et al. Paleointensity variations across the last geomagnetic reversal at La Palma, Canary Islands, Spain. *J. Geophys. Res. Solid Earth*. **104**, 7577–7598 (1999).

[28] Monster, M. W., de Groot, L. V., Biggin, A. J. & Dekkers, M. J. The performance of various palaeointensity techniques as a function of rock magnetic behaviour–A case study for La Palma. *Phys. Earth Planet. Inter*. **242**, 36–49 (2015).

[29] Calvo-Rathert, M. et al. Reliability of the palaeomagnetic signal recorded in a lava flow erupted on 4 December 2021 in La Palma (Canary Islands, Spain). *Geophys. J. Int.* **239**, 841–861 (2024).

[30] Paoletti, V., Secomandi, M., Fedi, M., Florio, G. & Rapolla, A. The integration of magnetic data in the Neapolitan volcanic district. *Geosphere* **1**, 85–96 (2005).

[31] Hinze, W. J., Von Frese, R. R., Von Frese, R. & Saad, A. H. *Gravity and Magnetic Exploration: Principles, practices, and Applications* (Cambridge University Press, 2013).

[32] Paoletti, V. et al. Subcircular conduits and dikes offshore the Somma-Vesuvius volcano revealed by magnetic and seismic data. *Geophys. Res. Lett.* **43**, 9544–9551 (2016).

[33] Dunlop, D. J. & Özdemir, Ö. *Rock Magnetism: Fundamentals and Frontiers* (Cambridge University Press, 1997).

[34] Soengkono, S. Interpretation of magnetic anomalies over the Waimangu geothermal area, Taupo volcanic zone, new Zealand. *Geothermics* **30**, 443–459 (2001).

[35] Carracedo, J. C., Pérez Torrado, F. J., Nuez, J. & Guillou, H. d. l. & Rodríguez Badiola, E. Geology of La Palma and El Hierro, Canary islands (2001).

[36] Staudigel, H. & Schmincke, H. Structural evolution of a seamount: evidence from the uplifted intraplate seamount on the Island of La Palma, Canary Islands. *Eos* **62**, 1075 (1981).

[37] Guillou, H., Carracedo, J. & Duncan, R. K–Ar, 40Ar–39Ar ages and magnetostratigraphy of Brunhes and Matuyama lava sequences from La Palma Island. *J. Volcanol Geotherm. Res.* **106**, 175–194 (2001).

[38] Guillou, H., Carracedo, J. C. & Day, S. J. Dating of the upper Pleistocene–Holocene volcanic activity of La Palma using the unspiked K–Ar technique. *J. Volcanol Geotherm. Res.* **86**, 137–149 (1998).

[39] Socías, I. & Mézcua, J. Levantamiento aeromagnético Del archipiélago Canario. *Instituto Geográfico Nac. Publicación Técnica No* 35 (1996).

[40] Quidelleur, X. & Valet, J. P. Geomagnetic changes across the last reversal recorded in lava flows from La Palma, Canary Islands. *J. Geophys. Res. Solid Earth*. **101**, 13755–13773 (1996).

[41] Quidelleur, X., Carlut, J., Gillot, P. Y. & Soler, V. Evolution of the geomagnetic field prior to the Matuyama—Brunhes transition: radiometric dating of a 820 ka excursion at La Palma. *Geophys. J. Int.* **151**, F6–F10 (2002).

[42] Secomandi, M. et al. Analysis of the magnetic anomaly field of the volcanic district of the Bay of Naples, Italy. *Mar. Geophys. Res.* **24**, 207–221 (2003).

[43] Paoletti, V., Rapolla, A. & Secomandi, M. Magnetic signature of submarine volcanoes in the phlegrean Fields-Ischia ridge (North-Western side of the Bay of Naples, Southern Italy). *Ann. Geophys.* **51** (2008).

[44] Austria, R. S. et al. Magnetic field characterization of Macolod corridor (Luzon, Philippines): new perspectives on rifting in a volcanic Arc setting. *Tectonophysics* **822**, 229179 (2022).

[45] Roest, W. R., Verhoef, J. & Pilkington, M. Magnetic interpretation using the 3-D analytic signal. *Geophysics* **57**, 116–125 (1992).

[46] Reid, A. B., Allsop, J., Granser, H., Millett, A. & Somerton, I. Magnetic interpretation in three dimensions using Euler Deconvolution. *Geophysics* **55**, 80–91 (1990).

[47] Reid, A. B., Ebbing, J. & Webb, S. J. Avoidable Euler errors–the use and abuse of Euler Deconvolution applied to potential fields. *Geophys. Prospect.* **62**, 1162–1168 (2014).

[48] Tanaka, A., Okubo, Y. & Matsubayashi, O. Curie point depth based on spectrum analysis of the magnetic anomaly data in East and Southeast Asia. *Tectonophysics* **306**, 461–470 (1999).

[49] Clark, D. A. Magnetic petrology of igneous intrusions: implications for exploration and magnetic interpretation. *Explor. Geophys.* **30**, 5–26 (1999).

[50] Singer, B. et al. Ar/Ar ages from transitionally magnetized lavas on La Palma, Canary Islands, and the geomagnetic instability timescale. *J. Geophys. Res. Solid Earth.* **107**, EPM 7-1-EPM 7–20 (2002).

[51] Coello, J. in *Simposio Internacional de Recursos Hidráulicos «Canarias Agua.*

[52] Masson, D. et al. Slope failures on the flanks of the Western Canary Islands. *Earth-Sci. Rev.* **57**, 1–35 (2002).

[53] Carbó, A., Muñoz-Martín, A., Llanes, P., Álvarez, J. & Group, E. W. Gravity analysis offshore the Canary Islands from a systematic survey. *Geophysics Canary Islands: Results Spain’s Exclusive Economic Zone Program* 113–127 (2005).

[54] Parés, J. M. et al. Rock Magnetism of Lapilli and Lava Flows from Cumbre Vieja Volcano, 2021 Eruption (La Palma, Canary Islands): Initial Reports. *Geosciences* **12**, 271 (2022).

[55] Padrón, E. et al. Helium emission at cumbre Vieja volcano, La Palma, Canary Islands. *Chem. Geol.* **312**, 138–147 (2012).

[56] Finn, C. A. et al. Geophysical imaging of the Yellowstone hydrothermal plumbing system. *Nature* **603**, 643–647 (2022). 35322248 10.1038/s41586-021-04379-1

