# Supplementary material for: Crustal structure of La Palma Island inferred from 3D aeromagnetic modelling
Source: Sci Rep. 2026 Jan 10;16:4513. doi: 10.1038/s41598-025-34611-1 (PMC12864949; doi:10.1038/s41598-025-34611-1)
Supplement: Supplementary file 1 — Supplementary Material 1 [file 41598_2025_34611_MOESM1_ESM.pdf]

Supplementary information

Crustal structure of La Palma Island inferred from 3D aeromagnetic modelling

María C. Romero-Toribio*[1,2], Fátima Martín-Hernández[1,2], Juanjo Ledo[1]

[1]Departamento de Física de la Tierra y Astrofísica, Facultad de Ciencias Físicas, Universidad Complutense de Madrid, Plaza de las Ciencias 1, 28040 Madrid, Spain.

[2]Instituto de Geociencias, IGEO (CSIC-UCM), Calle Doctor Severo Ochoa 7, 28040 Madrid, Spain.

*Corresponding author: María C. Romero-Toribio (mromer30@ucm.es)

Outline

- Flight lines in the aeromagnetic survey of 1993 in the Canary Archipelago (**Supplementary Figure S1**)
- Estimated depth to the bottom layer of the magnetic sources La Palma (**Supplementary Figure S2**)
- Error distribution from the standalone magnetic inversion (**Supplementary Figure S3**)
- **Supplementary Text**: details on the gravimetric and magnetic joint inversion
- Magnetic and gravimetric models from the joint inversion (**Supplementary Figure S4**)
- Magnetotelluric model from Di Paolo et al. (2020) (**Supplementary Figure S5**)

Flight lines in the aeromagnetic survey of 1993 in the Canary Archipelago

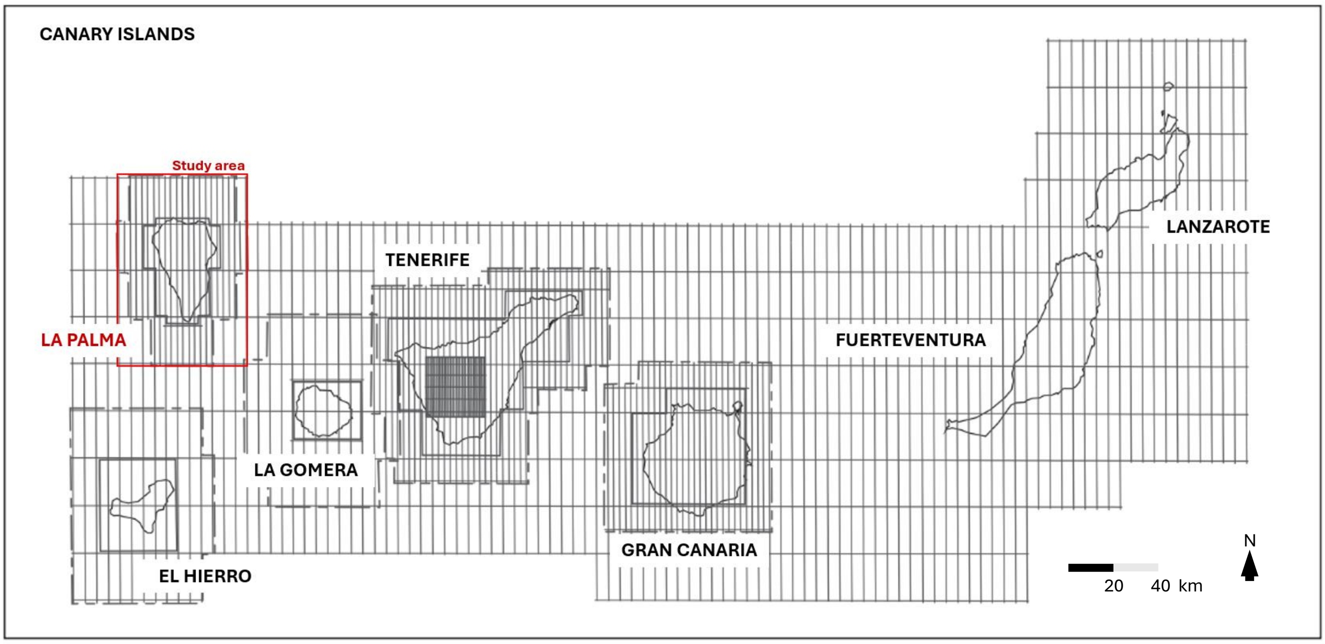

Figure S1. Flight line scheme of the aeromagnetic survey in the Canary Archipelago by the Spanish National Geographic Institute[1]. In La Palma, the N-S survey lines were spaced 2.5 km and the E-W tie lines were spaced 20 km. Figure modified after the original scheme in the Atlas Nacional de España[2]. This figure is covered by the Creative Commons Attribution 4.0 International License.

Estimated depth to the bottom layer of the magnetic sources La Palma

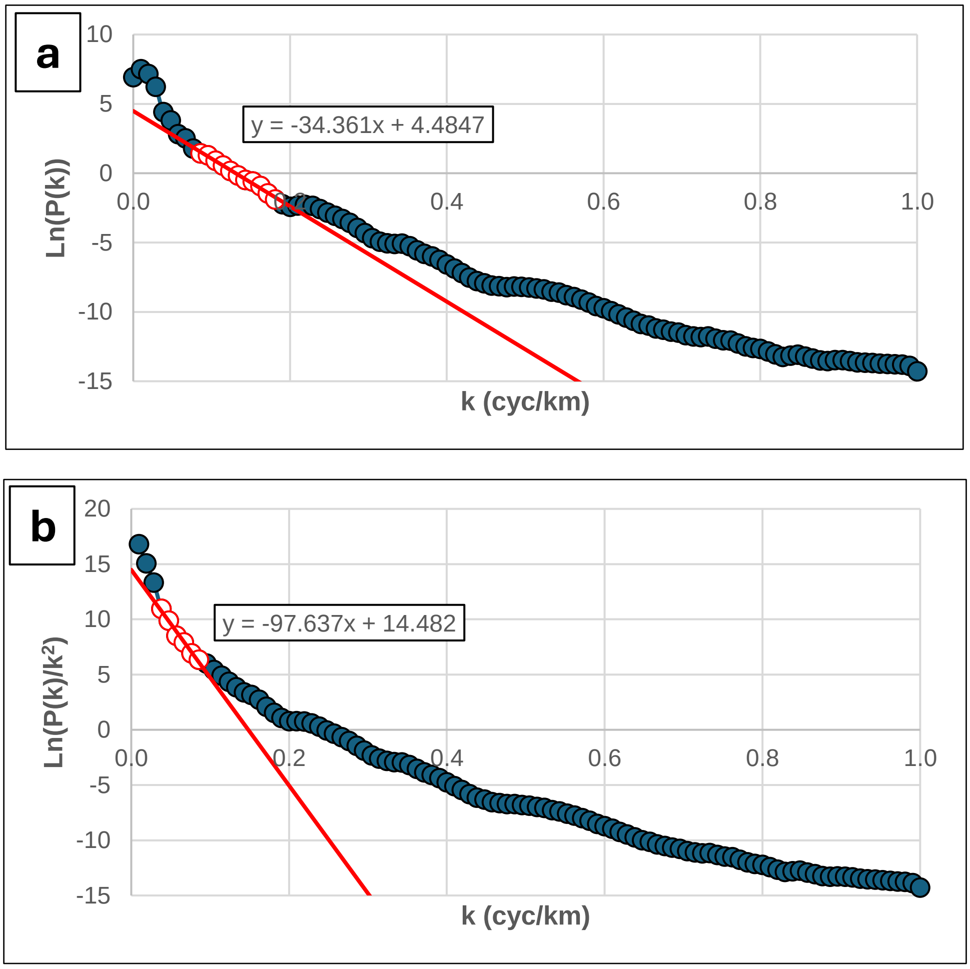

Zt	=	-0.47	±	0.19	km
Zo	=	4.6	±	1.4	km
Zb	=	9.6	±	2.8	km

Figure S2. Results from the centroid method to calculate the depth to the bottom of magnetic sources from the magnetic anomaly map across the entire island. a) Radially averaged power spectrum (P(k)) used to calculate the depth to the top of the magnetic layer (Zt). b) Radially averaged power spectrum scaled by the wavenumber ($P(k)/k^2$) used to calculate the depth to the centroid of the magnetic layer (Zo). The depth to the bottom (Zb) is calculated as Zb = 2Zo-Zt

Error distribution from the standalone magnetic inversion

The goodness of fit (GF) or error in the models was evaluated with Eq. [1]

$$GF_{(x,y)}(\%) = \left(\frac{M_{cal_{(x,y)}} - M_{obs_{(x,y)}}}{M_{obs_{(x,y)}}}\right) \cdot 100 \qquad \text{Eq. [1]}$$

where $M_{cal_{(x,y)}}$ is the calculated magnetic anomaly value from the susceptibility model assuming no significant remanent magnetization at the coordinate point (x, y) and $M_{obs_{(x,y)}}$ is the actual observed anomaly at each coordinate (x, y).

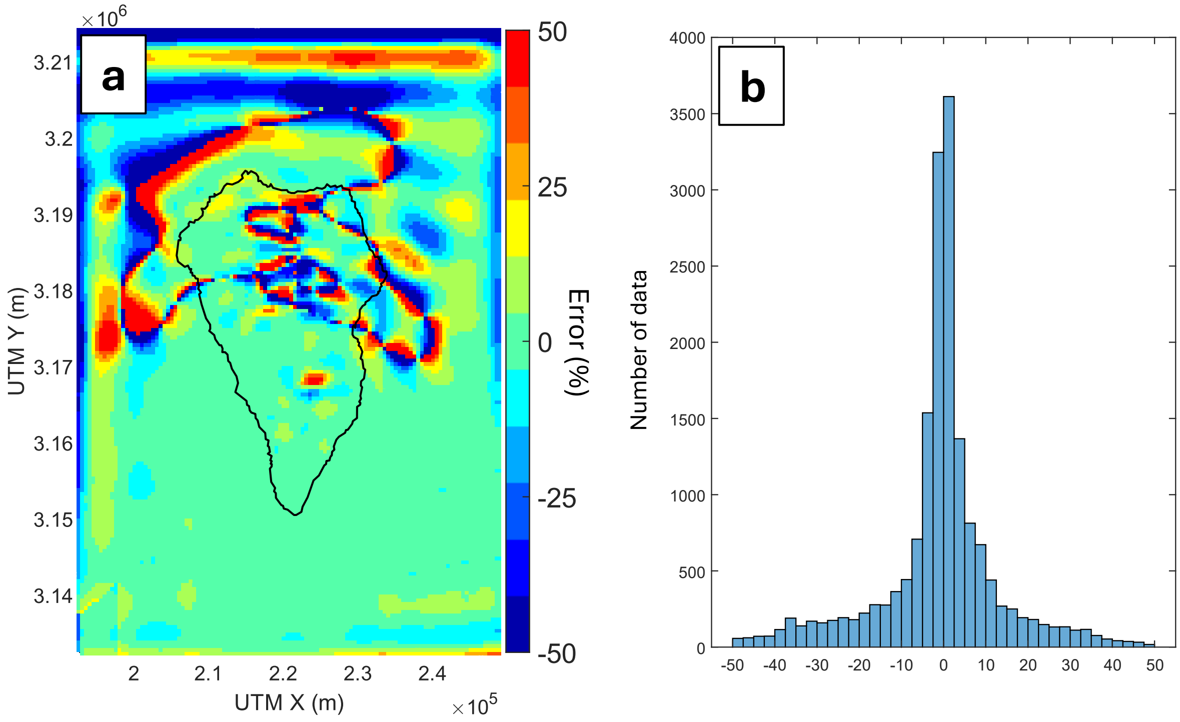

Figure S3. Error distributions derived from the standalone magnetic inversion

Supplementary Text: details on the gravimetric and magnetic joint inversion

The complete Bouguer anomaly dataset of La Palma as well as the elevation used in the joint modelling were obtained from Montesinos et al.[3] and the Spanish National Geographic Institute (IGN) levelling network (under request). The data was acquired in 2005 and 2021 (pre-Tajogaite eruption) comprising 498 sites. Details on data processing are available in Montesinos et al.[3]. By linearly fitting the anomaly with the topography in the N-S direction, we have determined a mean regional trend value of 61 ± 13 mGal/m which has been removed from the data to obtain the local Bouguer anomaly. Those gravity values range from approximately 29 mGal to -15 mGal and shows a decreasing tendency in the N-S direction.

We used the same software than the standalone magnetic model, ZondGM3D (http://zond-geo.com/english/). The joint inversion is calculated by means of the cross-gradients of both potential fields. To enable the calculation of the corresponding cross-gradients, the magnetic data (original grid) was linearly interpolated with MATLAB software (MATLAB Version R2024a) into the gravimetric site coordinates (498 data points). Therefore, the amount of magnetic data decreased from 18531 gridded data to 498 data points (remained 2.69 % of the original data) including the removal of offshore data. This led to a decrease in the horizontal resolution of the models.

The 3D initial model mesh for the inversion consisted of 42 × 77 horizontal cells and 14 vertical divisions increasing by a factor of 1.2, from 200 m a.s.l. for the subaerial part of the island to a bottom depth of 12 km bsl. The relief added to the initial mesh belongs to the gravimetric dataset (orthometric height). The depth constraints in the initial 1D model for the magnetic susceptibility model are analogue to the standalone magnetic inversion, including a susceptibility of $100 \cdot 10^{-4}$ (cgs) as half space value. The density contrast model was initiated at 0 g/cm^3 assuming the Bouguer anomaly reference value is 2.67 g/cm^3. We used the Occam method and 15 iterations with norm L2.

Magnetic and gravimetric models from the joint inversion

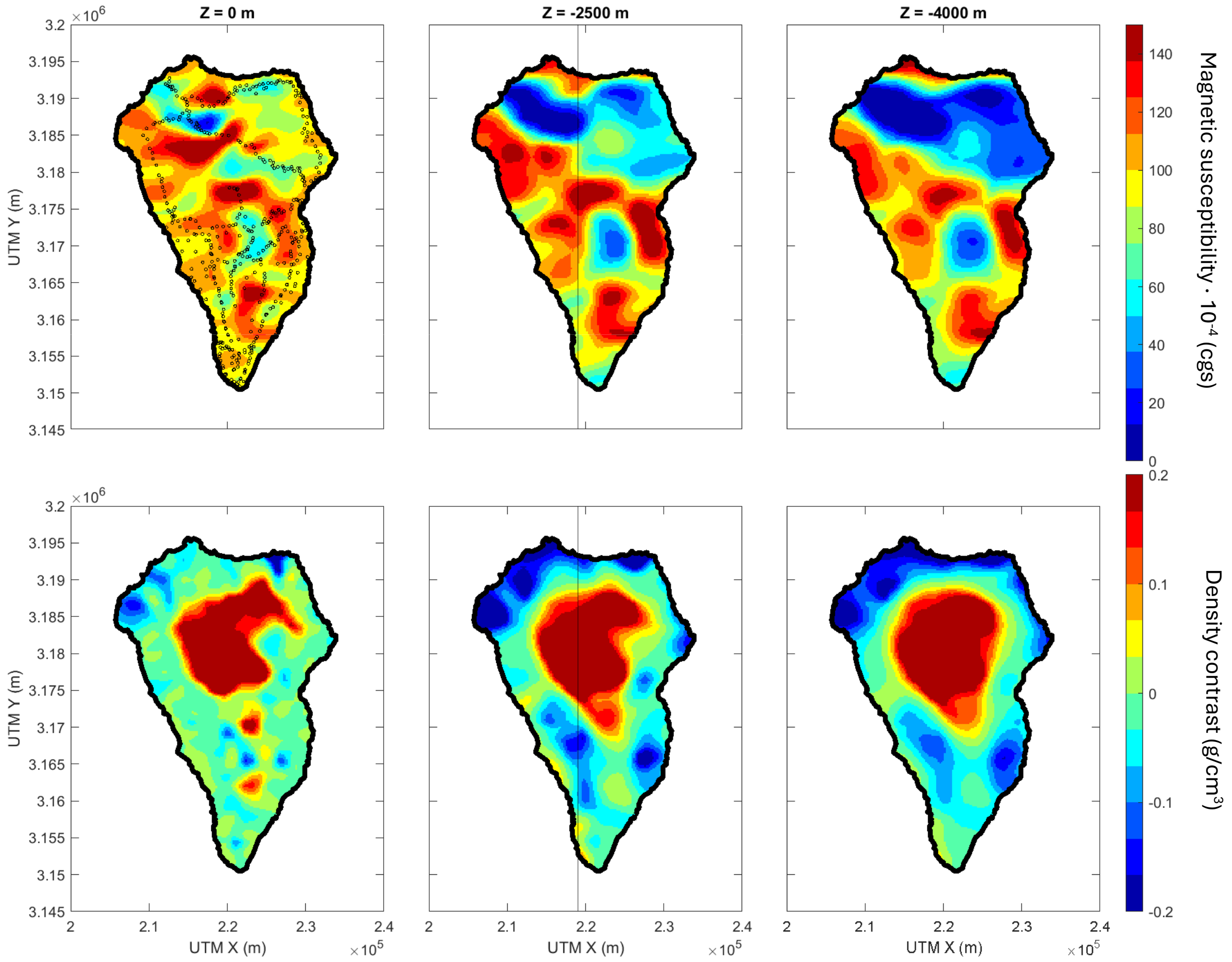

Figure S4. Magnetic susceptibility (up) and density contrast (down) models at three depths (sea level, 2500 m below sea level and 4000 m below sea level) derived from the joint inversion. Black circles indicate the input data distribution coincident with the gravimetric site coordinates. Solid black lines represent the cross sections (X = 219000 m) from Figure 4 in the manuscript.

Magnetotelluric model from Di Paolo et al.[4]

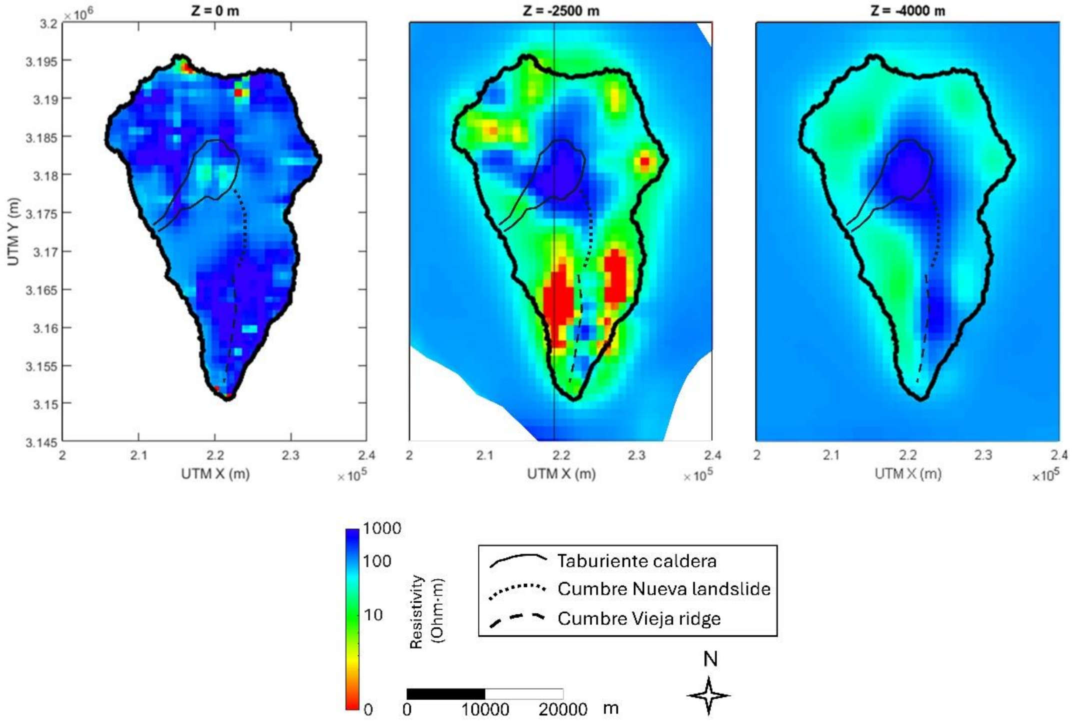

Figure S5. Magnetotelluric model from Di Paolo et al.[4] at three depths (sea level, 2500 m below sea level and 4000 m below sea level). Solid black line represents the cross section (X = 219000 m) from Figure 4 in the manuscript.

References

1. Socías, I. & Mézcua, J. Levantamiento aeromagnético del archipiélago canario. Instituto Geográfico Nacional, Publicación Técnica no 35 (1996).
2. Instituto Geográfico Nacional. Geofísica. 2nd ed. (2005). https://www.ign.es/web/resources/docs/IGNCnig/ANE/Publicacion/08_Geofisica_2005_2ed.pdf
3. Montesinos, F. G. *et al.* Insights into the Magmatic Feeding System of the 2021 Eruption at Cumbre Vieja (La Palma, Canary Islands) Inferred from Gravity Data Modeling. *Remote Sensing* **15**, 1936 (2023).
4. Di Paolo, F., *et al.* La Palma island (Spain) geothermal system revealed by 3D magnetotelluric data inversion. *Scientific reports*, *10*(1), 18181. (2020).
